# Skin Microbiota Diversity Is Associated with Biophysical Properties Across Healthy Human Skin Types

**DOI:** 10.3390/microorganisms14051026

**Published:** 2026-04-30

**Authors:** Ryosuke Kadoya, Ayano Kondo, Ayaka Matsukawa, Aoi Kuribayashi, Emi Uemura, Haruna Tanaka

**Affiliations:** Department of Food and Nutrition, School of Life Studies, Sugiyama Jogakuen University, 17-3 Hoshigaoka motomachi, Chikusa-ku, Nagoya 464-8662, Aichi, Japan; kaa20aa053@g.sugiyama-u.ac.jp (A.K.); msa20aa109@g.sugiyama-u.ac.jp (A.M.); kaa21aa053@g.sugiyama-u.ac.jp (A.K.); uea19aa014@g.sugiyama-u.ac.jp (E.U.); tha19aa070@g.sugiyama-u.ac.jp (H.T.)

**Keywords:** skin microbiota, healthy skin, microbial diversity

## Abstract

The skin microbiota plays a key role in maintaining cutaneous homeostasis; however, microbial differences among physiological skin types within healthy individuals remain unclear. In this study, we investigated the association between skin microbiota diversity and four skin types (normal, oily, dry, and combination) in healthy young women (*n* = 43), with samples collected from the nasal region. Skin moisture and sebum levels were quantitatively measured, and microbiota profiles were analyzed using 16S rRNA gene amplicon sequencing targeting the V3–V4 regions. Normal skin exhibited higher alpha diversity, including Chao1 richness and Faith’s phylogenetic diversity, compared with other skin types (median Chao1 values were higher in normal skin than in other groups). Correlation analyses showed that skin moisture was positively associated with microbial richness (ρ = 0.397, *p* = 0.008), whereas sebum levels were negatively associated with phylogenetic diversity (ρ = −0.455, *p* = 0.002). Beta diversity analysis revealed that normal skin harbored a distinct microbial community structure. In addition, several bacterial genera were enriched in normal skin, whereas Enterobacterales were observed to be more abundant in non-normal skin types. These findings suggest that skin biophysical properties are associated with microbial community structure and diversity within healthy individuals, although the functional implications of these differences remain to be elucidated.

## 1. Introduction

The skin is the largest organ of the human body, serving as the primary interface between the host and the external environment [1]. In addition to its well-established structural and immunological barrier functions, the skin harbors a diverse microbial ecosystem, collectively referred to as the skin microbiota, which contributes to skin physiology and health [2]. Recent studies have highlighted the pivotal role of skin microbiota in maintaining homeostasis by regulating immune responses, providing colonization resistance against pathogens, and supporting the integrity of the cutaneous barrier [3,4].

*Staphylococcus epidermidis* is one of the most well-characterized commensal bacteria on human skin. This species is known to metabolize sweat and sebum components to produce antimicrobial fatty acids and glycerol, thereby contributing to barrier function and the defense against pathogenic microbes [5,6]. The fatty acids produced by *S. epidermidis* help maintain the mildly acidic pH of the skin, which can inhibit colonization by pathogenic microorganisms, whereas glycerol contributes to skin hydration and epidermal barrier integrity [2,7]. Collectively, these metabolites are thought to play important roles in skin health. Accordingly, *S. epidermidis* is generally regarded as a beneficial commensal bacterium that contributes to cutaneous homeostasis and protection from pathogens [2,8].

Another major commensal bacterium of the human skin, *Cutibacterium acnes* (formerly *Propionibacterium acnes*), predominantly inhabits hair follicles and sebaceous glands, where it metabolizes sebum to produce short-chain fatty acids, including propionic acid. These metabolic byproducts contribute to the maintenance of a mildly acidic skin surface, which can suppress colonization by pathogenic microorganisms [2,3]. However, the dysregulated or excessive proliferation of *C. acnes* has been implicated in inflammatory responses, most notably in acne vulgaris [9]. Together, these phenomena highlight the context-dependent roles of cutaneous commensals and underscore that skin health is shaped not by the presence of individual taxa alone but by the balanced activity of the microbial community, influencing barrier function, immune modulation, and resistance to pathogenic colonization [4,10].

Advances in next-generation sequencing, particularly in 16S rRNA gene profiling, have expanded our understanding of cutaneous microbial diversity [10,11]. Studies have revealed that the skin microbiome comprises a broad range of bacterial taxa beyond the well-characterized species *S. epidermidis* and *C. acnes*. However, their functional roles, interactions, and ecological contributions of many of these taxa are poorly understood [10]. Addressing this knowledge gap is essential to develop a more comprehensive understanding of how microbial communities shape skin physiology and pathology.

Previous research has largely adopted a dichotomous framework in which skin conditions are classified as either “disease states”, such as atopic dermatitis or psoriasis, or “healthy states” [10,12,13]. Although this approach has provided valuable insights, it overlooks the substantial heterogeneity that exists in clinically healthy skin. Therefore, in the present study, we focused exclusively on healthy skin and further stratified it into four categories—normal, oily, dry, and combination skin—based on quantitative measurements of skin moisture and sebum content [14,15,16]. Normal skin represents a physiologically balanced condition, whereas oily, dry, and combination skin reflect distinct imbalances in hydration and lipid production. By analyzing the skin microbiome across these categories, this study sought to clarify how differences in microbial community composition are associated with distinct skin health states, thereby advancing the current understanding of the relationship between cutaneous microbial ecosystems and skin physiology.

## 2. Materials and Methods

### 2.1. Measurement of Skin Moisture and Sebum Content

Measurement of the skin moisture and sebum content, as well as the collection of skin bacteria, was conducted in healthy female subjects aged 21–22 years (*n* = 43). Participants were classified into four skin types based on quantitative measurements, and the number of participants in each group was as follows: normal (*n* = 19), oily (*n* = 13), dry (*n* = 5), and combination (*n* = 6). The measurement site was the nasal bridge, and measurements were conducted between May and June. The nasal bridge was selected as the sampling site because it represents a sebaceous area with relatively high microbial density and is commonly used in skin microbiome studies to capture variations associated with skin physiology. Written informed consent was obtained from all participants prior to enrollment, and the study was approved by the Ethics Committee of Sugiyama Jogakuen University (approval number: 2022-2 and 2023-17).

Measurements were performed in a controlled indoor environment maintained at 22–25 °C. On the day of the measurement, participants refrained from using cosmetics or skincare products. The participants were healthy individuals without known dermatological conditions. The exclusion criteria included recent antibiotic use, dermatological conditions, and ongoing topical or systemic treatments affecting skin physiology. Skin moisture and sebum content were measured using the Triple Sense device (Moritex, Kanagawa, Japan). Skin type classification was performed based on the moisture and sebum levels according to the manufacturer’s recommended thresholds, which are commonly used in cosmetic and clinical applications for standardized skin assessment. Each parameter was measured in triplicate and averaged for analysis, with standard deviations generally within 5–10% of the mean, indicating acceptable reproducibility.

### 2.2. Quantification of Skin Commensal Bacteria

Skin commensal bacteria were collected and cultured using the following procedure. The nasal bridge area (1 × 2 cm^2^) was swabbed with a sterile cotton swab (J.C.B. Industry Limited, Tokyo, Japan) moistened with sterile water, while applying consistent pressure. After collection, the cotton swabs were immersed in 2 mL of sterile water and thoroughly vortexed to prepare a suspension of skin commensal bacteria. The resulting suspension was serially diluted (10^−1^ to 10^−3^) with sterile water, and 100 µL of each dilution was spread onto nutrient agar and GAM agar plates. The culture medium was incubated aerobically or anaerobically at 37 °C for 48 h. Anaerobic culture was performed using an AnaeroPack system and an anaerobic jar (Sugiyama Gen, Tokyo, Japan). After incubation, the number of colonies formed was counted, and the bacterial abundance was expressed as colony-forming units per square centimeter (CFU/cm^2^).

### 2.3. Preparation of Samples for 16S rRNA Gene Amplicon Sequencing

Genomic DNA was extracted from bacterial pellets using the DNeasy^®^ PowerBiofilm Kit (Qiagen, Hilden, North Rhine-Westphalia, Germany) according to the manufacturer’s instructions. The extracted DNA was eluted in autoclaved distilled water, and DNA concentration and purity were assessed using a NanoDrop™ 2000 spectrophotometer (Thermo Scientific, Waltham, MA, USA).

The skin microbiota were profiled using 16S rRNA gene amplicon sequencing targeting the V3–V4 hypervariable regions. PCR amplification was performed using the primer pair 314F (5′-ACACTCTTTCCCTACACGACGCTCTTCCGATCT-NNNNN-CCTACGGGNGGCWGCAG-3′) and 805R (5′-GTGACTGGAGTTCAGACGTGTGCTCTTCCGATCT-NNNNN-GACTACHVGGGTATCTAATCC-3′). PCR was carried out using TaKaRa Ex Taq^®^ Hot Start Version (TaKaRa, Kyoto, Japan) on a Takara PCR Thermal Cycler Dice Gradient under the following conditions: initial denaturation at 95 °C for 3 min; 30 cycles of denaturation at 95 °C for 30 s, annealing at 55 °C for 30 s, and extension at 72 °C for 60 s; followed by a final extension at 72 °C for 10 min.

Sequencing libraries were normalized based on DNA concentration, pooled, and sequenced on an Illumina MiSeq platform using a 2 × 300 bp paired-end V3–V4 sequencing kit. The average sequencing depth was approximately 27,500 reads per sample (range: 18,652–38,119 reads), and no samples were excluded after quality control.

Negative controls were included during DNA extraction and PCR amplification and were processed alongside all samples to monitor potential contamination. No amplification was observed in these controls.

### 2.4. Microbial Community Analysis

Microbial community analysis was conducted using QIIME 2 software (version 2021.11) [17]. Demultiplexed paired-end sequences were imported into QIIME 2, and quality filtering, denoising, and chimera removal were performed using the DADA2 denoise-paired plugin, resulting in the generation of amplicon sequence variants (ASVs).

Alpha and beta diversity analyses were performed on rarefied data with a sampling depth of 18,000 reads per sample. Alpha diversity was assessed using the Chao1 richness estimator and the Shannon diversity index. Beta diversity was evaluated based on unweighted UniFrac distances and visualized using principal coordinate analysis (PCoA). Taxonomic classification was performed using a naïve Bayes classifier trained on the SILVA SSU rRNA database (release 138), trimmed to the V3–V4 region. Sequences were assigned to taxonomic levels up to the genus level where possible. On average, 94.9% of reads were assigned at the genus level (range: 73.8–100%), indicating a high level of taxonomic resolution. The proportion of unclassified reads was low, and no major unclassified groups were observed.

Differentially abundant taxa between skin types were identified using linear discriminant analysis effect size (LEfSe) implemented in the Galaxy web platform (https://huttenhower.sph.harvard.edu/lefse/ (accessed on 12 December 2025)) [18], with a logarithmic LDA score threshold of >2.0. Cladograms were generated based on the LEfSe results.

### 2.5. Statistical Analysis

All statistical analyses were performed using EZR (version 1.68; Saitama Medical Center, Jichi Medical University, Tochigi, Japan) and R software (version 4.5.2; R Foundation for Statistical Computing, Vienna, Austria) [19].

Differences among groups were assessed using the Kruskal–Wallis test. When significant differences were detected, post hoc pairwise comparisons were performed using the Steel–Dwass test, which accounts for multiple comparisons. Correlation analyses were conducted using Spearman’s rank correlation coefficient.

Statistical significance was set at *p* < 0.05, and 0.05 ≤ *p* < 0.10 was considered to indicate a trend.

## 3. Results and Discussion

### 3.1. Relationship Between Skin Biophysical Properties and Microbial Abundance Across Skin Types

**Figure 1:**
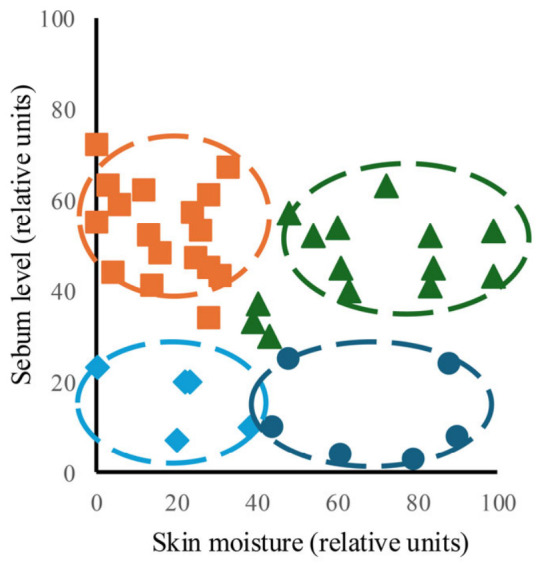
Distribution of skin moisture and sebum content across different skin types. The vertical axis represents the sebum content, and the horizontal axis represents the moisture content. Values on both axes are shown as relative units. Orange squares indicate normal skin, green triangles indicate oily skin, light blue diamonds indicate dry skin, and navy circles indicate combination skin. Each skin type is displayed according to the classification provided by the Triple Sense device. Group sizes were as follows: normal (*n* = 19), oily (*n* = 13), dry (*n* = 5), and combination (*n* = 6).

First, each particpaint’s skin type was determined based on the quantitative measurements of skin moisture and sebum content obtained from the nasal area, and the results are shown in Figure 1. Individual data points for sebum and moisture content were plotted for each participant, revealing a distinct pattern of moisture and sebum levels for each of the four skin types: normal skin (moisture, 41–67; sebum, 0–46), oily skin (moisture, 43–62; sebum, 61–99), dry skin (moisture, 7–23; sebum, 0–23), and combination skin (moisture, 3–24; sebum, 44–90).

**Figure 2:**
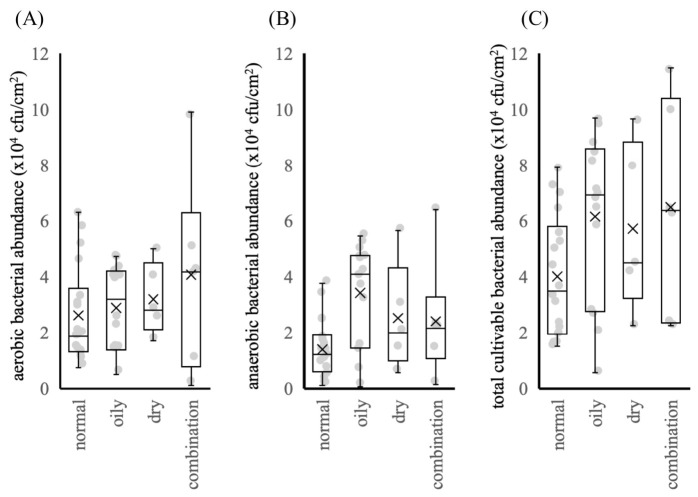
Cultivable bacterial abundance across different skin types. The vertical axis represents the total bacterial count (CFU/cm^2^). (**A**) Aerobic bacterial counts, (**B**) anaerobic bacterial counts, and (**C**) total bacterial counts. Data are presented as the median with interquartile range, and individual data points are shown. The cross indicates the mean value. The number of participants in each group was as follows: normal (*n* = 19), oily (*n* = 13), dry (*n* = 5), and combination (*n* = 6). Statistical analysis was performed using the Kruskal–Wallis test followed by the Steel–Dwass test. Statistical significance was defined as *p* < 0.05; however, no significant differences were observed between the groups.

Skin bacteria were collected from the nasal area of each participant using sterile swabs. The samples were inoculated onto standard nutrient agar plates and cultured under aerobic conditions, as well as onto GAM agar plates and cultured under anaerobic conditions. The aerobic and anaerobic bacterial counts are shown separately in Figure 2A,B, respectively, and their sum was used to estimate the total cultivable bacterial load (Figure 2C). The bacterial counts for each skin type ranged as follows: normal skin, 1.6–7.9 × 10^4^ CFU/cm^2^; oily skin, 0.6–9.7 × 10^4^ CFU/cm^2^; dry skin, 5.2–9.7 × 10^4^ CFU/cm^2^; and combination skin, 2.3–10.0 × 10^4^ CFU/cm^2^. No statistically significant differences in bacterial counts were observed among the skin types, althought the bacterial counts in oily, dry, and combination skin were numerically higher than those in normal skin. Previous studies have reported that increased bacterial colonization of the skin is associated with deteriorated skin conditions [20]. Accordingly, the modest differences in bacterial counts observed between normal skin, which is considered to be in a more balanced condition, and the other skin types may reflect subtle variations in skin condition.

### 3.2. Comparative Analysis of Skin Microbiota Composition Among Different Skin Types

**Figure 3:**
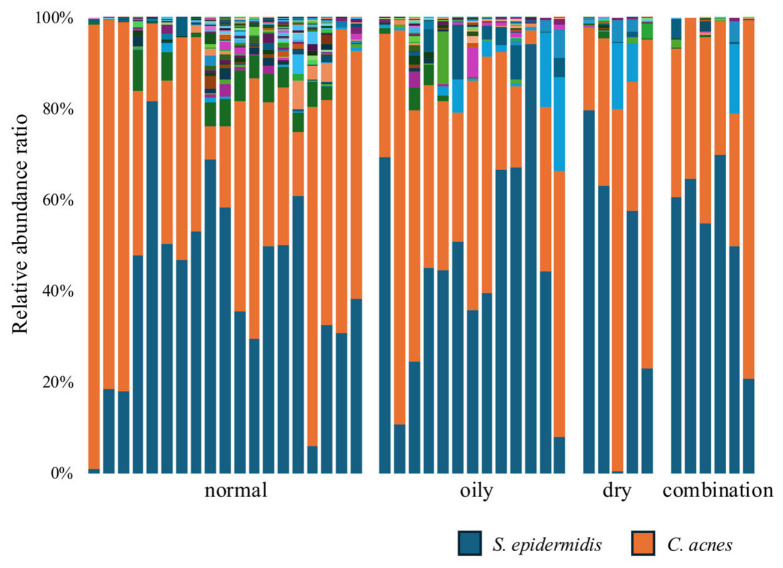
Relative abundance of skin bacterial communities at the genus level across different skin types. Each color represents the relative abundance of a bacterial taxon in the stacked bar chart. *S. epidermidis* is shown in blue, and *C. acnes* is shown in orange. Other colors represent bacterial genera present at lower relative abundances. Each bar represents the skin microbiota of an individual participant. Group sizes were as follows: normal (*n* = 19), oily (*n* = 13), dry (*n* = 5), and combination (*n* = 6).

**Figure 4:**
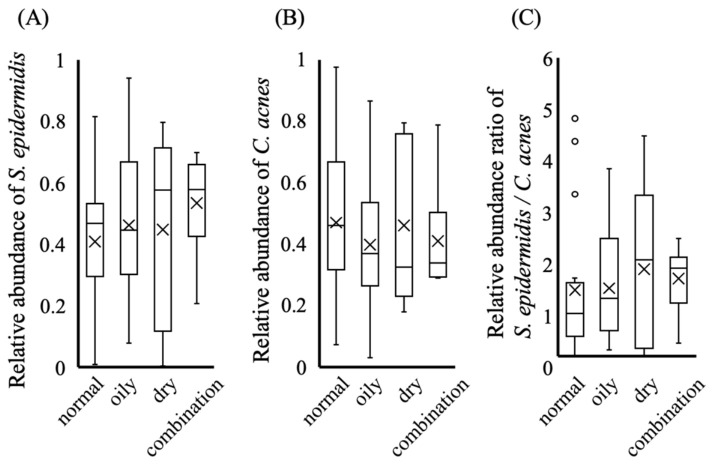
Relative abundance and bacterial counts across different skin types. (**A**) Relative abundance of *S. epidermidis*, (**B**) relative abundance of *C. acnes*, (**C**) ratio of *S. epidermidis* to *C. acnes*. Data are presented as the median with interquartile range, and individual data points are shown. The cross indicates the mean value. The number of participants in each group was as follows: normal (*n* = 19), oily (*n* = 13), dry (*n* = 5), and combination (*n* = 6). Statistical analysis was performed using the Kruskal–Wallis test followed by the Steel–Dwass test. No significant differences were observed between the groups (*p* > 0.05).

Chromosomal DNA was extracted from bacteria collected from the nasal skin of each participant, and the skin microbiota were analyzed by sequencing the V3–V4 region of the 16S rRNA gene. The microbiota of each skin type are shown in Figure 3. Before the experiment, we hypothesized that normal skin would exhibit a high relative abundance of *S. epidermidis* and a low relative abundance of *C. acnes*. In contrast, other skin types were expected to show a lower abundance of *S. epidermidis* and a higher abundance of *C. acnes*. However, the results did not support this hypothesis. Figure 3 shows no significant differences in the relative abundances of *S. epidermidis* (blue) and *C. acnes* (orange) among the different skin types. For a more detailed comparison, the distributions of *S. epidermidis* and *C. acnes* among the skin types are depicted as box-and-whisker plots in Figure 4A,B. Subsequently, the balance between *S. epidermidis* and *C. acnes* was considered a potential factor contributing to skin health (Figure 4C). In all comparisons, no significant differences were observed among the skin types.

Previous studies comparing healthy skin with disease states, such as atopic dermatitis, have reported marked differences in the abundance of key commensal taxa, including *Staphylococcus* and *Cutibacterium*, suggesting that shifts in these bacteria are closely linked to pathological conditions [21,22]. In contrast, the present study focused exclusively on clinically healthy skin and further subdivided it into four skin types based on their biophysical properties. Within this framework, neither the relative abundance of *S. epidermidis* nor that of *C. acnes* differed considerably among the skin types examined. These findings suggest that, although alterations in these taxa may serve as meaningful indicators of disease-associated dysbiosis, they may have limited utility in discriminating physiological variations within healthy skin. Thus, the differences in skin type between healthy individuals are likely shaped not by the dominance of individual bacterial species but rather by more subtle changes in the overall community structure, diversity, or the presence of less abundant taxa.

### 3.3. Characterization of Skin Microbiota Diversity in Relation to Skin Type

**Figure 5:**
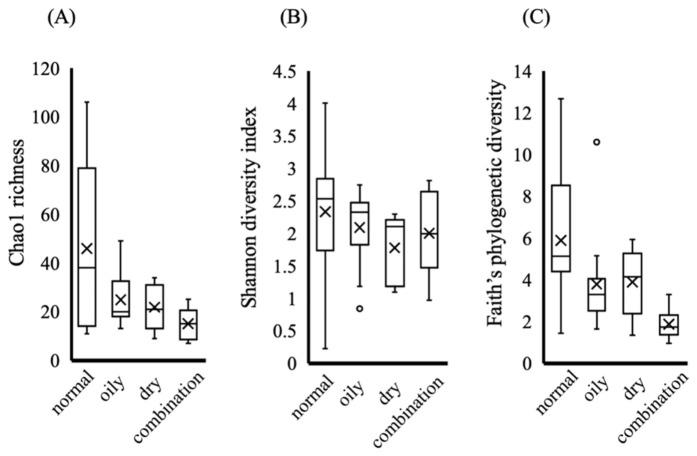
Alpha diversity metrics across different skin types. (**A**) Chao1 richness, (**B**) Shannon index, and (**C**) Faith’s phylogenetic diversity. Boxes represent the interquartile range, with the median indicated by a horizontal line. Whiskers indicate the minimum and maximum values. Data are presented as median with interquartile range, and individual data points are shown. The cross indicates the mean value. Group sizes were as follows: normal (*n* = 19), oily (*n* = 13), dry (*n* = 5), and combination (*n* = 6). Statistical analysis was performed using the Kruskal–Wallis test followed by Dunn’s multiple comparison test for pairwise comparisons. Significant differences identified by post hoc analysis are described in the main text.

The influence of skin type (normal, oily, dry, and combination) on the microbial diversity was evaluated using multiple alpha diversity metrics (Figure 5A–C).

Chao1 richness differed significantly among skin types overall (Kruskal–Wallis test, *p* = 0.028). Normal skin showed the highest median values for this richness-based index; however, pairwise comparisons did not reveal statistically significant differences between individual groups. In contrast, no significant differences were observed in the Shannon diversity index (*p* = 0.283), suggesting that overall community evenness may be relatively stable across skin types. As the Shannon index reflects both richness and evenness, this result suggests that differences among skin types were not strongly driven by variation in evenness. Notably, the phylogenetic diversity (Faith’s PD) showed significant differences among groups (*p* = 0.004), and pairwise comparisons indicated that combination skin exhibited significantly lower diversity than normal (*p* = 0.022) and oily skin (*p* = 0.042). Taken together, these results indicate that the differences in skin microbiota among skin types may be more prominently reflected in phylogenetic diversity than in species richness or evenness. Although richness-based metrics suggested a tendency toward higher diversity in normal skin, the lack of statistical significance in pairwise comparisons indicates that these differences should be interpreted cautiously. This pattern may reflect limited statistical power due to the subgroup size imbalance.

Previous studies on dermatological conditions such as atopic dermatitis have reported that lesional skin exhibits reduced microbial diversity relative to healthy control skin, consistent with a pattern of microbial dysbiosis in disease states [23,24]. In contrast, the present study focused on variation within clinically healthy individuals and suggests that differences among physiological skin types may be more subtle and primarily reflected in community structure rather than dominant taxa or overall evenness. Skin biophysical parameters, including the sebum content and moisture levels, are considered key environmental factors shaping the microbial community structure by creating distinct ecological niches across the skin surface [2]. Previous studies have demonstrated that variation in sebum and hydration levels significantly influence the composition and diversity of the skin microbiome in healthy individuals [25]. In addition, recent studies have begun to examine associations between microbiome composition and physiological skin characteristics in healthy populations, although such investigations remain limited [26]. However, most previous studies have focused on site-specific differences rather than systematic comparisons between physiological skin types within a homogeneous population. These findings suggest that microbial diversity may be associated with physiologically balanced skin conditions; however, the functional implications of these differences remain to be elucidated. Furthermore, the differences in diversity metrics such as Chao1 richness or the number of detected taxa do not necessarily translate into functional or clinical differences in the skin microbiota. Importantly, as this study is based on a cross-sectional design, these associations should not be interpreted as evidence of a causal relationship. It remains unclear whether higher microbial diversity contributes to balanced skin conditions or is a consequence of them.

**Figure 6:**
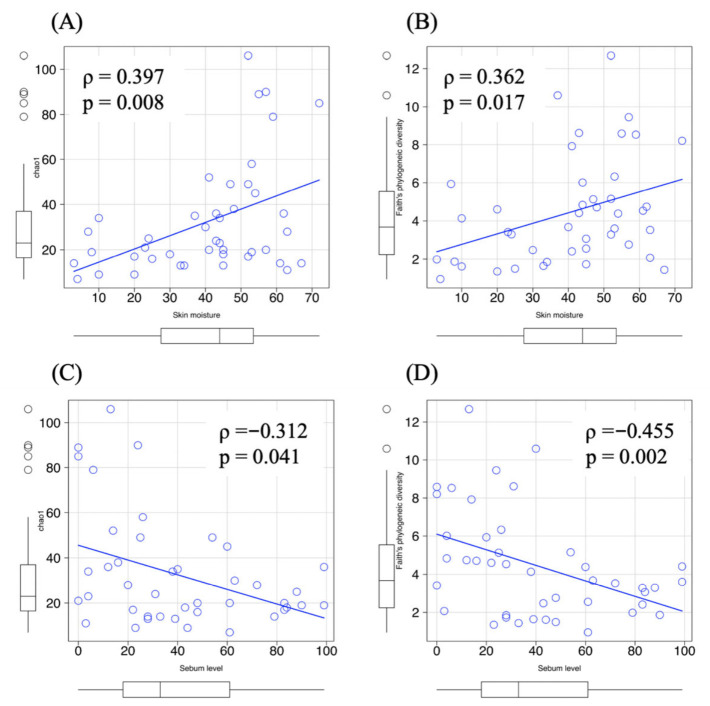
Associations between skin biophysical parameters and alpha diversity of the skin microbiota. (**A**) Relationship between skin moisture and Chao1 richness. (**B**) Relationship between sebum level and Chao1 richness. (**C**) Relationship between skin moisture and Faith’s phylogenetic diversity. (**D**) Relationship between sebum level and Faith’s phylogenetic diversity. Correlation analysis was performed using Spearman’s rank correlation. Spearman’s rank correlation coefficients (ρ) and corresponding *p*-values are shown in each panel. Solid lines represent linear regression fits with 95% confidence intervals. Group sizes were as follows: normal (*n* = 19), oily (*n* = 13), dry (*n* = 5), and combination (*n* = 6).

To further explore whether these differences were associated with skin biophysical parameters, we performed correlation analyses. Spearman’s rank correlation analysis showed that skin moisture was positively associated with microbial richness (Chao1; ρ = 0.397, *p* = 0.008) and phylogenetic diversity (Faith’s PD; ρ = 0.362, *p* = 0.017) (Figure 6A,B). In contrast, sebum levels were negatively associated with Chao1 richness (ρ = −0.312, *p* = 0.041) and showed a moderate negative association with Faith’s PD (ρ = −0.455, *p* = 0.002) (Figure 6C,D). No significant correlations were observed between moisture or sebum levels and Shannon diversity (*p* > 0.05).

These results suggest that microbial diversity may be associated primarily with taxonomic richness and phylogenetic breadth rather than overall community evenness. Higher moisture levels may be associated with the coexistence of a broader range of taxa, whereas elevated sebum levels may act as an ecological filter that favors lipid-adapted lineages and reduces phylogenetic diversity without markedly altering dominance structure.

**Figure 7:**
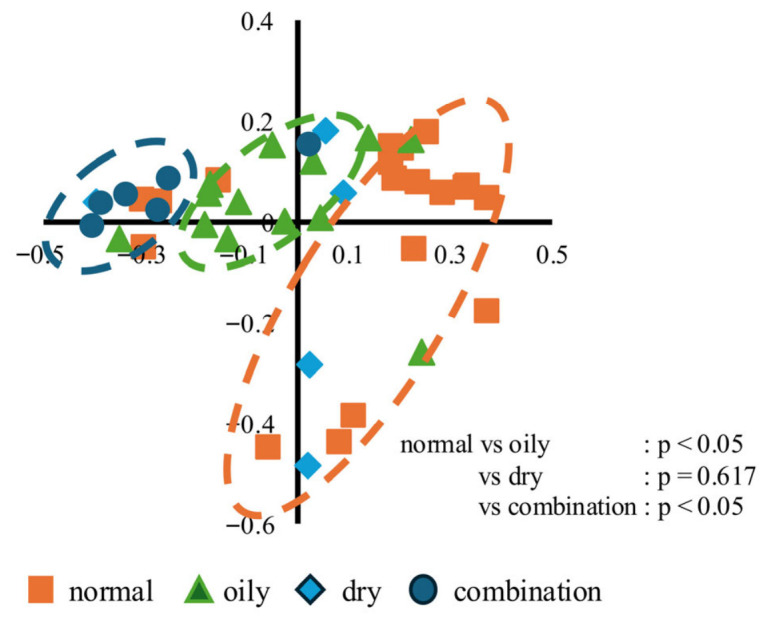
Principal coordinate analysis (PCoA) based on unweighted UniFrac distances showing the beta diversity of the skin microbiota across different skin types. Orange denotes normal skin, green denotes oily skin, light blue denotes dry skin, and blue denotes combination skin. PC1 explains 22.27% of the variation, and PC2 explains 12.33% of the variation. Statistical differences between groups were assessed using PERMANOVA, and significant pairwise differences between normal skin and other skin types (*p* < 0.05) are indicated in the figure. Group sizes were as follows: normal (*n* = 19), oily (*n* = 13), dry (*n* = 5), and combination (*n* = 6).

Beta diversity, calculated using unweighted UniFrac distances, was analyzed using principal coordinate analysis (PCoA), which revealed differences in the community structure among skin types (Figure 7). Normal, oily, and combination skin types showed partially distinct clustering patterns of bacterial communities. Pairwise comparisons of community similarity using PERMANOVA demonstrated significant differences between normal and oily skin, as well as between normal and combination skin, indicating low similarity in their microbial community compositions (*p* < 0.05). In contrast, no significant differences were observed between normal and dry skin, which may be attributable to the relatively small sample size. Together with the observed differences in alpha diversity, these beta diversity patterns indicate that normal skin harbors a relatively distinct microbial ecosystem compared to other skin types, which is consistent with previous reports demonstrating shifts in the skin microbial community structure between healthy and disease states [10,20].

### 3.4. Linear Discriminant Analysis Effect Size (LEfSe)-Based Identification of Skin Type-Associated Microbial Taxa

**Figure 8:**
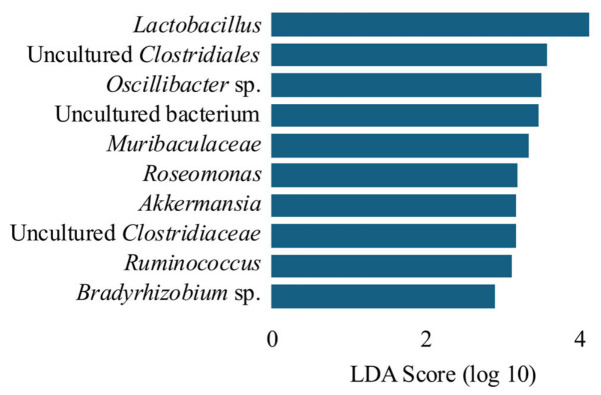
Linear discriminant analysis effect size (LEfSe) identifying bacterial genera differentially enriched in normal skin compared with other skin types. Genera with *p* < 0.05 (Kruskal–Wallis test) and an LDA score > 2 were considered significant and are shown. Group sizes were as follows: normal (*n* = 19), oily (*n* = 13), dry (*n* = 5), and combination (*n* = 6).

Because differences in the dominant taxa alone did not explain the observed variations in alpha and beta diversity, we next applied LEfSe to identify the microbial taxa associated with normal skin compared with other skin types (Figure 8). The results showed that 10 bacterial genera were identified as enriched in normal skin. Among these 10 bacterial genera, four have been previously reported to be associated with skin conditions, namely, *Lactobacillus*, *Roseomonas*, *Ruminococcus*, and *Kocuria* spp. Members of the genus *Lactobacillus* have been reported to be associated with beneficial effects on skin health, particularly through the application of bacterial lysates or fermentation products (postbiotics) [27,28]. Several studies have reported that the topical application of *Lactobacillus*-derived products, such as bacterial lysates or fermentation lysates (postbiotics), can improve skin barrier function and health [29,30]. *Roseomonas* spp. are skin-resident bacteria. In a previous study, *Roseomonas* spp. isolated from healthy skin were applied to patients with atopic dermatitis, resulting in an improvement in symptoms [31]. *Ruminococcus* spp. are mucinolytic enterobacteria. Oral administration of *Ruminococcus* spp. in mice with atopic dermatitis has been reported to improve their condition [32]. Members of the genus *Kocuria*, which are commonly found as part of the normal skin microbiota, have been associated with skin environments that may inhibit inflammation and pathogen overgrowth, consistent with the observation that commensal skin bacteria can help resist pathogen colonization [33]. These taxa may represent candidate microbial markers of balanced skin physiology; however, functional validation studies are required.

Notably, although not identified as a significant feature in the LEfSe, members of the order *Enterobacterales* were abundant in oily, dry, and combination skin types. Compared with normal skin, their relative abundance was 13.7-fold higher in oily skin, 14.8-fold higher in dry skin, and 8.4-fold higher in combination skin. *Enterobacterales* are typically low-abundance or opportunistic members of the healthy skin microbiota. Therefore, their increased relative abundance in oily, dry, and combination skin types may reflect subtle alterations in the cutaneous microbial environment associated with imbalances in sebum and moisture. Similar patterns have been reported in some dermatological conditions where microbial dysbiosis is characterized by shifts in low-abundance taxa [34,35]. The enrichment of *Enterobacterales* in non-normal skin types may reflect differences in the skin microenvironment; however, any association with sebum and moisture imbalance remains speculative and should be interpreted as a hypothesis.

The observed differences in skin microbiota associated with normal skin may be influenced by biophysical factors such as moisture and sebum levels, which are known to modulate microbial colonization and growth [2,36]. Alternatively, specific bacterial taxa enriched in normal skin may be associated with skin homeostasis, potentially through mechanisms such as the competitive exclusion of pathogens, modulation of local immune responses, or the production of antimicrobial compounds [6,22]. As these findings were derived from bioinformatics-based analyses, further studies involving the isolation and functional characterization of these bacterial genera from normal skin are warranted. Collectively, the results of this study provide a foundation for elucidating the relationship between skin health and skin microbiota and may facilitate the identification of microbial factors that contribute to the maintenance of skin homeostasis.

## 4. Conclusions

In this study, 16S rRNA gene amplicon sequencing was used to investigate the relationship between skin microbiota diversity and four distinct healthy skin types: normal, oily, dry, and combination skin. The microbial richness and phylogenetic diversity were significantly higher in normal skin, which is often considered to represent a more physiologically balanced state of the four types. Correlation analyses demonstrated that skin moisture was positively associated with microbial richness and phylogenetic diversity, whereas sebum levels were negatively associated with phylogenetic diversity. These findings suggest that skin biophysical properties are linked to the phylogenetic structure of the skin microbiota and may act as environmental filters shaping microbial community composition within healthy individuals. Furthermore, this analysis identified bacterial taxa predominantly associated with the normal skin type. Future studies integrating metagenomics, cultivation, and functional assays will be required to elucidate the ecological and physiological roles of these taxa in maintaining skin homeostasis.

This study has several limitations that should be considered when interpreting the findings. The cohort was relatively small and homogeneous, consisting only of healthy young women within a narrow age range, and samples were collected from a single anatomical site during a limited seasonal window. As the nasal area is characteristically sebaceous, the extent to which these measurements reflect whole-face skin types, particularly combination skin, may be limited. These factors may limit the generalizability of the findings to other populations, including different age groups, sexes, ethnic backgrounds, and anatomical sites. Potential confounding factors such as diet, hormonal status, and environmental exposure were also not fully controlled. Furthermore, a swab-based negative control was not included during sample collection, which may limit the assessment of potential environmental contamination. Despite these limitations, the present study provides novel insights into microbiome variation across physiological skin types in healthy individuals and highlights the role of skin biophysical properties in shaping microbial community structure.

## Data Availability

The original contributions presented in this study are included in the article. Further inquiries can be directed to the corresponding author.
